# 4-[(*E*)-2-(Pyridin-2-yl)ethen­yl]pyridine–terephthalic acid (2/1)

**DOI:** 10.1107/S1600536812046284

**Published:** 2012-11-17

**Authors:** Paola Castro-Montes, Jorge A. Guerrero-Alvarez, Herbert Hopfl, Jose J. Campos-Gaxiola, Adriana Cruz-Enriquez

**Affiliations:** a Facultad de Ingenieria Mochis, Universidad Autonoma de Sinaloa, Fuente Poseidon y Prol. A. Flores S/N, CP 81223, C.U. Los Mochis, Sinaloa, Mexico; b Centro de Investigaciones Quimicas, Universidad Autonoma del Estado de Morelos, Av. Universidad 1001, CP 62210, Cuernavaca, Morelos, Mexico

## Abstract

The title 2:1 co-crystal, 2C_12_H_10_N_2_·C_8_H_6_O_4_, crystallizes with one mol­ecule of 4-[(*E*)-2-(pyridin-2-yl)ethen­yl]pyridine (*A*) and one half-mol­ecule of terephthalic acid (*B*) in the asymmetric unit. In the crystal, the components are linked through heterodimeric COOH⋯N_pyridine_ synthons, forming linear aggregates of composition –*A*–*B*–*A*–*B*–. Further linkage through weak C—H⋯O and C—H⋯π inter­actions gives two-dimensional hydrogen-bonded undulating sheets propagating in the [100] and [010] directions. These layers are connected through additional weak C—H⋯O contacts, forming a three-dimensional structure.

## Related literature

For reports on supra­molecular crystal engineering and potential applications of co-crystals, see: Desiraju (1995 ▶); Simon & Bassoul (2000 ▶); Bhogala & Nangia (2003 ▶); Weyna *et al.* (2009 ▶); Yan *et al.* (2012 ▶). For background to related co-crystals, see: Santra *et al.* (2008 ▶); Moon & Park (2012 ▶); Ebenezer & Muthiah (2012 ▶).
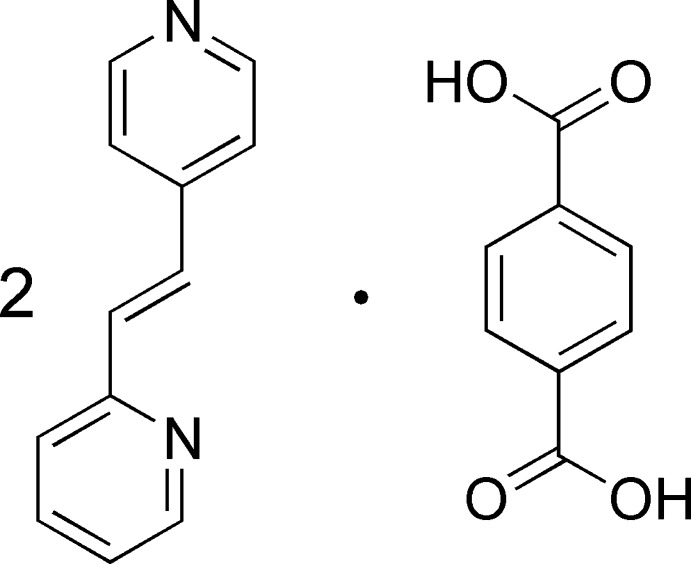


## Experimental

### 

#### Crystal data

C_12_H_10_N_2_·0.5C_8_H_6_O_4_
*M* *_r_* = 265.28
Monoclinic, 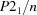

*a* = 6.3821 (8) Å
*b* = 32.301 (4) Å
*c* = 6.8721 (8) Å
β = 111.440 (2)°
*V* = 1318.6 (3) Å^3^
*Z* = 4
Mo *K*α radiation
μ = 0.09 mm^−1^
*T* = 293 K
0.48 × 0.41 × 0.34 mm

#### Data collection

Bruker SMART CCD area-detector diffractometer
Absorption correction: multi-scan (*SADABS*; Sheldrick, 1996 ▶) *T*_min_ = 0.96, *T*_max_ = 0.97
12715 measured reflections
2328 independent reflections
2119 reflections with *I* > 2σ(*I*)
*R*_int_ = 0.033

#### Refinement

*R*[*F* ^2^ > 2σ(*F* ^2^)] = 0.055
*wR*(*F* ^2^) = 0.149
*S* = 1.17
2328 reflections
184 parameters
1 restraint
H atoms treated by a mixture of independent and constrained refinement
Δρ_max_ = 0.14 e Å^−3^
Δρ_min_ = −0.20 e Å^−3^

### 

Data collection: *SMART* (Bruker, 2000 ▶); cell refinement: *SAINT-Plus* (Bruker 2001 ▶); data reduction: *SAINT-Plus*; program(s) used to solve structure: *SHELXS97* (Sheldrick, 2008 ▶); program(s) used to refine structure: *SHELXL97* (Sheldrick, 2008 ▶); molecular graphics: *ORTEP-3* (Farrugia, 2012 ▶) and *Mercury* (Macrae *et al.* 2008 ▶); software used to prepare material for publication: *publCIF* (Westrip, 2010 ▶).

## Supplementary Material

Crystal structure: contains datablock(s) I, global. DOI: 10.1107/S1600536812046284/su2525sup1.cif

Structure factors: contains datablock(s) I. DOI: 10.1107/S1600536812046284/su2525Isup2.hkl

Supplementary material file. DOI: 10.1107/S1600536812046284/su2525Isup3.cml

Additional supplementary materials:  crystallographic information; 3D view; checkCIF report

## Figures and Tables

**Table 1:** Hydrogen-bond geometry (Å, °) *Cg* is the centroid of the N2/C12–C16 pyridine ring.

*D*—H⋯*A*	*D*—H	H⋯*A*	*D*⋯*A*	*D*—H⋯*A*
O1—H1′⋯N1^i^	0.84	1.77	2.604 (2)	177
C9—H9⋯O2^ii^	0.93	2.67	3.285 (3)	125
C13—H13⋯O2^iii^	0.93	2.52	3.396 (2)	157
C5—H5⋯O2^iv^	0.93	2.64	3.135 (2)	114
C16—H16⋯*Cg* ^v^	0.93	2.86	3.627 (3)	141

Symmetry codes: (i) 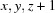; (ii) 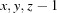; (iii) 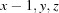; (iv) 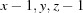; (v) 
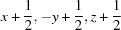
.

**Fig. 1.**
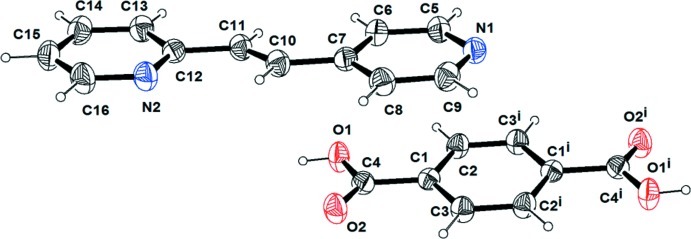
The molecular structures of the components in the title compound, showing the atom numbering. Displacement ellipsoids are drawn at the 50% probability level. [symmetry code: (i) -x + 2, -y, -z + 1].

**Fig. 2.**
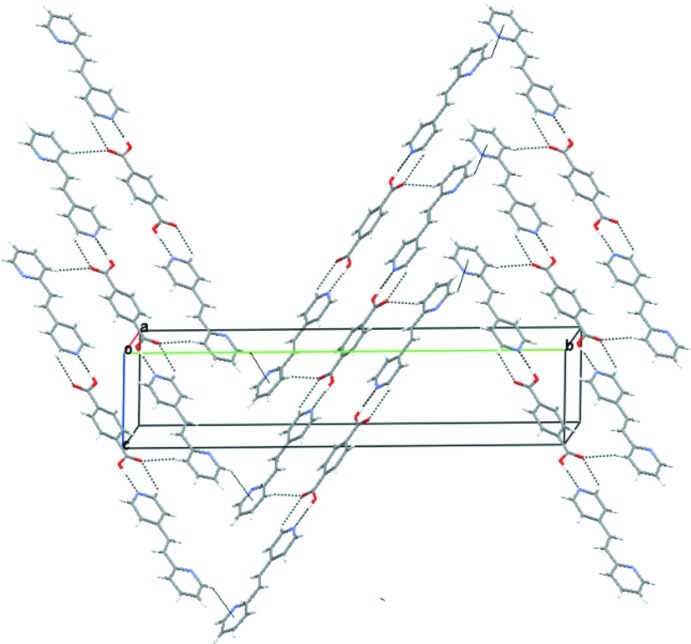
View of the two-dimensional supramolecular layer formed through O—H···N, C—H···O and C—H···π interactions (dashed lines; see Table 1 for details), in the crystal structure of the title compound.

## supplementary crystallographic information

### Comment

Supramolecular crystal engineering has attracted growing interest over the past few decades because of its importance in biological systems, molecular recognition (Simon *et al.*, 2000), pharmaceutical chemistry (Weyna *et al.*, 2009) and materials chemistry (Yan *et al.*, 2012). Aromatic carboxylic acids form reliable supramolecular synthons for the construction of novel organic networks by hydrogen bonding and π–π interactions (Desiraju, 1995), and numerous studies have focused on hydrogen bonding between carboxylic acids and pyridine molecules (Bhogala & Nangia, 2003; Santra *et al.*, 2008; Moon & Park, 2012; Ebenezer & Muthiah, 2012). Herein, we report on the solid-state structure of a 2:1 co-crystal formed between an asymmetric bipyridine [4-((*E*)-2-(pyridin-2-yl)ethenyl)pyridine] and a symmetric dicarboxylic acid [terephthalic acid].

The molecular structure of the title compound is shown in Fig. 1. The asymmetric unit contains one molecule of 4-((*E*)-2-(pyridin-2-yl)ethenyl)pyridine and half a molecule of terephthalic acid located on a crystallographic inversion center. Both components have almost planar molecular structures as seen from the C10—C11—C12—N2 torsion angle of -4.2 (3)° for the bipyridine molecule and the O1—C4—C1—C2 torsion angle of -6.0 (3)° for the terephthalic acid.

In the crystal lattice, each terephthalic acid is linked to two bipyridine molecules through intermolecular O—H···N and C—H···O interactions giving the well known heterodimeric COOH···N_pyridine_ synthon. The so formed linear aggregates are connected through additional weak C—H···O contacts to generate tapes parallel to the (1–41) series of planes, which through C—H···π contacts generate undulating two-dimensional supramolecular layers (Fig. 2 and Table 1). In the third dimension, these layers are interconnected through additional weak C—H···O contacts. Interestingly, the 2-pyridine nitrogen atom is not involved in short intermolecular hydrogen bonding interactions.

### Experimental

0.200 g (1.10 mmol) of 4-((*E*)-2-(pyridin-2-yl)ethenyl)pyridine and 0.180 g (1.10 mmol) of terephthalic acid were ground in a mortar for 20 min after adding 3 drops of CH_3_OH. The resulting powder was then dissolved in 10 ml of CH_3_OH and kept for crystallization by slow evaporation of the solvent at ambient conditions to give colourless block-like crystals, suitable for single-crystal X-ray diffraction analysis, after one week. Spectroscopic and TGA data for the title compound are available in the archived CIF.

### Refinement

H atoms bonded to C atoms were positioned geometrically and constrained using the riding-model approximation [aryl C—H = 0.93 A and *U*_iso_(H) = 1.2*U*_eq_(C)]. The H atom bonded to O was initially located in a difference Fourier map, then the position was refined with the O—H distance restraint of 0.84 (1) Å with *U*_iso_(*H*) = 1.5*U*_eq_(O). One reflection that was located behind the beam stop has been omitted during the refinement (020).

### Crystal data

**Table:**

C_12_H_10_N_2_·0.5C_8_H_6_O_4_	*F*(000) = 556
*M**_r_* = 265.28	*D*_x_ = 1.336 Mg m^−^^3^
Monoclinic, *P*2_1_/*n*	Mo *K*α radiation, λ = 0.71073 Å
Hall symbol: -P 2yn	Cell parameters from 4971 reflections
*a* = 6.3821 (8) Å	θ = 2.5–27.1°
*b* = 32.301 (4) Å	µ = 0.09 mm^−^^1^
*c* = 6.8721 (8) Å	*T* = 293 K
β = 111.440 (2)°	Block, colourless
*V* = 1318.6 (3) Å^3^	0.48 × 0.41 × 0.34 mm
*Z* = 4	

### Data collection

**Table:**

Bruker SMART CCD area-detector diffractometer	2328 independent reflections
Radiation source: fine-focus sealed tube	2119 reflections with *I* > 2σ(*I*)
Graphite monochromator	*R*_int_ = 0.033
phi and ω scans	θ_max_ = 25.0°, θ_min_ = 2.5°
Absorption correction: multi-scan (*SADABS*; Sheldrick, 1996)	*h* = −7→7
*T*_min_ = 0.96, *T*_max_ = 0.97	*k* = −38→38
12715 measured reflections	*l* = −8→8

### Refinement

**Table:**

Refinement on *F*^2^	Primary atom site location: structure-invariant direct methods
Least-squares matrix: full	Secondary atom site location: difference Fourier map
*R*[*F*^2^ > 2σ(*F*^2^)] = 0.055	Hydrogen site location: inferred from neighbouring sites
*wR*(*F*^2^) = 0.149	H atoms treated by a mixture of independent and constrained refinement
*S* = 1.17	*w* = 1/[σ^2^(*F*_o_^2^) + (0.0769*P*)^2^ + 0.2452*P*] where *P* = (*F*_o_^2^ + 2*F*_c_^2^)/3
2328 reflections	(Δ/σ)_max_ < 0.001
184 parameters	Δρ_max_ = 0.14 e Å^−^^3^
1 restraint	Δρ_min_ = −0.20 e Å^−^^3^

### Special details

**Table:**

Experimental. Spectroscopic and TGA data for the title compound: IR (KBr): 3056, 2944, 1706, 1683, 1606, 1581, 1504, 1425, 1290 *y* 731 cm^-1^. ^1^H-RMN (200 MHz, DMSO-*d*_6_, TMS): *δ* 8.59 (m, 3H), 8.04 (s, 4H), 7.83 (td, *J* = 0.8, 4 Hz, 1H), 7.61 (m, 5H), 7.32 (m, 1H). TGA Calcd. for 2 C_12_H_10_N_2_: 68.69. Found: 69.27% (303 - 533 K).
Geometry. All e.s.d.'s (except the e.s.d. in the dihedral angle between two l.s. planes) are estimated using the full covariance matrix. The cell e.s.d.'s are taken into account individually in the estimation of e.s.d.'s in distances, angles and torsion angles; correlations between e.s.d.'s in cell parameters are only used when they are defined by crystal symmetry. An approximate (isotropic) treatment of cell e.s.d.'s is used for estimating e.s.d.'s involving l.s. planes.
Refinement. Refinement of *F*^2^ against ALL reflections. The weighted *R*-factor *wR* and goodness of fit *S* are based on *F*^2^, conventional *R*-factors *R* are based on *F*, with *F* set to zero for negative *F*^2^. The threshold expression of *F*^2^ > σ(*F*^2^) is used only for calculating *R*-factors(gt) *etc*. and is not relevant to the choice of reflections for refinement. *R*-factors based on *F*^2^ are statistically about twice as large as those based on *F*, and *R*- factors based on ALL data will be even larger.

### Fractional atomic coordinates and isotropic or equivalent isotropic displacement parameters (Å^2^)

**Table:**

	*x*	*y*	*z*	*U*_iso_*/*U*_eq_	
O1	0.7604 (3)	0.03567 (5)	0.8732 (2)	0.0626 (4)	
H1'	0.752 (4)	0.0508 (7)	0.969 (3)	0.094*	
O2	1.0571 (2)	0.07552 (4)	0.9137 (2)	0.0646 (4)	
C1	0.9678 (3)	0.02216 (5)	0.6602 (3)	0.0419 (4)	
C2	0.8138 (3)	−0.00755 (6)	0.5490 (3)	0.0497 (5)	
H2	0.6876	−0.0128	0.5824	0.060*	
C3	1.1549 (3)	0.02934 (6)	0.6094 (3)	0.0502 (5)	
H3	1.2603	0.0491	0.6830	0.060*	
C4	0.9339 (3)	0.04711 (6)	0.8289 (3)	0.0478 (5)	
N1	0.7178 (3)	0.08162 (5)	0.1678 (2)	0.0525 (4)	
N2	0.6102 (3)	0.20912 (5)	0.9682 (3)	0.0577 (5)	
C5	0.5534 (3)	0.07399 (6)	0.2373 (3)	0.0525 (5)	
H5	0.4559	0.0521	0.1784	0.063*	
C6	0.5203 (3)	0.09677 (6)	0.3915 (3)	0.0515 (5)	
H6	0.4020	0.0903	0.4346	0.062*	
C7	0.6625 (3)	0.12946 (6)	0.4837 (3)	0.0471 (5)	
C8	0.8332 (3)	0.13732 (7)	0.4096 (3)	0.0567 (5)	
H8	0.9330	0.1590	0.4651	0.068*	
C9	0.8553 (4)	0.11300 (7)	0.2539 (3)	0.0580 (5)	
H9	0.9714	0.1188	0.2067	0.070*	
C10	0.6360 (3)	0.15554 (6)	0.6476 (3)	0.0519 (5)	
H10	0.7328	0.1781	0.6911	0.062*	
C11	0.4900 (3)	0.15052 (6)	0.7397 (3)	0.0509 (5)	
H11	0.3948	0.1277	0.6996	0.061*	
C12	0.4641 (3)	0.17767 (5)	0.8998 (3)	0.0472 (5)	
C13	0.2917 (3)	0.17128 (6)	0.9726 (3)	0.0545 (5)	
H13	0.1941	0.1490	0.9238	0.065*	
C14	0.2652 (4)	0.19789 (7)	1.1171 (3)	0.0628 (6)	
H14	0.1481	0.1942	1.1654	0.075*	
C15	0.4136 (4)	0.22994 (7)	1.1891 (3)	0.0656 (6)	
H15	0.4008	0.2484	1.2880	0.079*	
C16	0.5813 (4)	0.23397 (7)	1.1110 (4)	0.0682 (6)	
H16	0.6827	0.2557	1.1613	0.082*	

### Atomic displacement parameters (Å^2^)

**Table:**

	*U*^11^	*U*^22^	*U*^33^	*U*^12^	*U*^13^	*U*^23^
O1	0.0692 (9)	0.0688 (10)	0.0642 (9)	−0.0138 (7)	0.0414 (8)	−0.0204 (7)
O2	0.0679 (9)	0.0625 (9)	0.0677 (9)	−0.0156 (7)	0.0300 (8)	−0.0246 (7)
C1	0.0449 (10)	0.0379 (9)	0.0434 (10)	0.0019 (7)	0.0166 (8)	0.0039 (7)
C2	0.0455 (10)	0.0521 (11)	0.0588 (11)	−0.0072 (8)	0.0276 (9)	−0.0060 (9)
C3	0.0508 (11)	0.0474 (10)	0.0553 (11)	−0.0111 (8)	0.0228 (9)	−0.0094 (8)
C4	0.0498 (10)	0.0485 (11)	0.0452 (10)	0.0027 (8)	0.0174 (8)	0.0011 (8)
N1	0.0618 (10)	0.0534 (9)	0.0457 (9)	0.0075 (8)	0.0237 (8)	0.0007 (7)
N2	0.0646 (11)	0.0492 (9)	0.0602 (10)	−0.0050 (8)	0.0238 (8)	−0.0120 (8)
C5	0.0594 (12)	0.0499 (11)	0.0494 (11)	−0.0002 (9)	0.0215 (9)	−0.0047 (8)
C6	0.0560 (11)	0.0508 (11)	0.0521 (11)	−0.0006 (9)	0.0250 (9)	−0.0039 (8)
C7	0.0525 (11)	0.0440 (10)	0.0443 (10)	0.0072 (8)	0.0170 (8)	0.0036 (8)
C8	0.0592 (12)	0.0568 (12)	0.0559 (11)	−0.0050 (9)	0.0232 (10)	−0.0035 (9)
C9	0.0620 (12)	0.0632 (13)	0.0576 (12)	0.0011 (10)	0.0323 (10)	0.0025 (10)
C10	0.0596 (11)	0.0439 (10)	0.0522 (11)	−0.0025 (8)	0.0205 (9)	−0.0047 (8)
C11	0.0594 (11)	0.0433 (10)	0.0499 (11)	−0.0015 (9)	0.0199 (9)	−0.0059 (8)
C12	0.0555 (11)	0.0399 (10)	0.0439 (10)	0.0051 (8)	0.0154 (8)	0.0020 (7)
C13	0.0643 (12)	0.0461 (11)	0.0548 (11)	0.0005 (9)	0.0239 (10)	0.0014 (8)
C14	0.0770 (14)	0.0600 (13)	0.0610 (12)	0.0113 (11)	0.0367 (11)	0.0038 (10)
C15	0.0885 (16)	0.0541 (12)	0.0562 (12)	0.0142 (11)	0.0288 (11)	−0.0072 (10)
C16	0.0810 (15)	0.0527 (12)	0.0669 (13)	−0.0062 (11)	0.0224 (12)	−0.0188 (10)

### Geometric parameters (Å, º)

**Table:**

O1—C4	1.305 (2)	C7—C8	1.384 (3)
O1—H1'	0.8401 (10)	C7—C10	1.465 (3)
O2—C4	1.210 (2)	C8—C9	1.376 (3)
C1—C3	1.381 (2)	C8—H8	0.9300
C1—C2	1.386 (3)	C9—H9	0.9300
C1—C4	1.491 (3)	C10—C11	1.314 (3)
C2—C3^i^	1.371 (3)	C10—H10	0.9300
C2—H2	0.9300	C11—C12	1.463 (3)
C3—C2^i^	1.371 (3)	C11—H11	0.9300
C3—H3	0.9300	C12—C13	1.380 (3)
N1—C5	1.325 (2)	C13—C14	1.369 (3)
N1—C9	1.330 (3)	C13—H13	0.9300
N2—C16	1.332 (3)	C14—C15	1.368 (3)
N2—C12	1.342 (2)	C14—H14	0.9300
C5—C6	1.369 (3)	C15—C16	1.368 (3)
C5—H5	0.9300	C15—H15	0.9300
C6—C7	1.386 (3)	C16—H16	0.9300
C6—H6	0.9300		
			
C4—O1—H1'	108.9 (19)	C7—C8—H8	120.0
C3—C1—C2	118.76 (17)	N1—C9—C8	122.86 (19)
C3—C1—C4	119.37 (16)	N1—C9—H9	118.6
C2—C1—C4	121.86 (16)	C8—C9—H9	118.6
C3^i^—C2—C1	120.91 (17)	C11—C10—C7	126.94 (18)
C3^i^—C2—H2	119.5	C11—C10—H10	116.5
C1—C2—H2	119.5	C7—C10—H10	116.5
C2^i^—C3—C1	120.33 (17)	C10—C11—C12	125.68 (18)
C2^i^—C3—H3	119.8	C10—C11—H11	117.2
C1—C3—H3	119.8	C12—C11—H11	117.2
O2—C4—O1	124.02 (17)	N2—C12—C13	122.00 (17)
O2—C4—C1	122.08 (17)	N2—C12—C11	117.51 (17)
O1—C4—C1	113.89 (16)	C13—C12—C11	120.47 (17)
C5—N1—C9	117.55 (16)	C14—C13—C12	119.7 (2)
C16—N2—C12	116.65 (18)	C14—C13—H13	120.2
N1—C5—C6	123.07 (18)	C12—C13—H13	120.2
N1—C5—H5	118.5	C15—C14—C13	119.0 (2)
C6—C5—H5	118.5	C15—C14—H14	120.5
C5—C6—C7	120.20 (18)	C13—C14—H14	120.5
C5—C6—H6	119.9	C14—C15—C16	117.86 (19)
C7—C6—H6	119.9	C14—C15—H15	121.1
C8—C7—C6	116.35 (17)	C16—C15—H15	121.1
C8—C7—C10	120.33 (18)	N2—C16—C15	124.8 (2)
C6—C7—C10	123.31 (17)	N2—C16—H16	117.6
C9—C8—C7	119.96 (19)	C15—C16—H16	117.6
C9—C8—H8	120.0		
			
C3—C1—C2—C3^i^	−0.4 (3)	C7—C8—C9—N1	0.2 (3)
C4—C1—C2—C3^i^	178.17 (17)	C8—C7—C10—C11	−176.30 (19)
C2—C1—C3—C2^i^	0.4 (3)	C6—C7—C10—C11	5.1 (3)
C4—C1—C3—C2^i^	−178.21 (17)	C7—C10—C11—C12	−178.50 (17)
C3—C1—C4—O2	5.2 (3)	C16—N2—C12—C13	0.2 (3)
C2—C1—C4—O2	−173.37 (18)	C16—N2—C12—C11	178.75 (18)
C3—C1—C4—O1	−175.43 (17)	C10—C11—C12—N2	−4.2 (3)
C2—C1—C4—O1	6.0 (3)	C10—C11—C12—C13	174.40 (19)
C9—N1—C5—C6	0.0 (3)	N2—C12—C13—C14	0.8 (3)
N1—C5—C6—C7	−0.3 (3)	C11—C12—C13—C14	−177.69 (18)
C5—C6—C7—C8	0.5 (3)	C12—C13—C14—C15	−1.2 (3)
C5—C6—C7—C10	179.21 (18)	C13—C14—C15—C16	0.5 (3)
C6—C7—C8—C9	−0.5 (3)	C12—N2—C16—C15	−0.9 (3)
C10—C7—C8—C9	−179.21 (18)	C14—C15—C16—N2	0.6 (4)
C5—N1—C9—C8	0.1 (3)		

Symmetry code: (i) −*x*+2, −*y*, −*z*+1.

### Hydrogen-bond geometry (Å, º)

*Cg* is the centroid of the N2/C12–C16 pyridine ring.

**Table:**

*D*—H···*A*	*D*—H	H···*A*	*D*···*A*	*D*—H···*A*
O1—H1′···N1^ii^	0.84	1.77	2.604 (2)	177
C9—H9···O2^iii^	0.93	2.67	3.285 (3)	125
C13—H13···O2^iv^	0.93	2.52	3.396 (2)	157
C5—H5···O2^v^	0.93	2.64	3.135 (2)	114
C16—H16···*Cg*^vi^	0.93	2.86	3.627 (3)	141

Symmetry codes: (ii) *x*, *y*, *z*+1; (iii) *x*, *y*, *z*−1; (iv) *x*−1, *y*, *z*; (v) *x*−1, *y*, *z*−1; (vi) *x*+1/2, −*y*+1/2, *z*+1/2.
